# Acute administration of methylphenidate differentially affects cortical processing of emotional facial expressions in attention-deficit hyperactivity disorder children as studied by functional near-infrared spectroscopy

**DOI:** 10.1117/1.NPh.7.2.025003

**Published:** 2020-05-06

**Authors:** Megumi Kobayashi, Takahiro Ikeda, Tatsuya Tokuda, Yukifumi Monden, Masako Nagashima, Sakae G. Mizushima, Takeshi Inoue, Keiichi Shimamura, Yuta Ujiie, Akari Arakawa, Chie Kuroiwa, Mayuko Ishijima, Yuki Kishimoto, So Kanazawa, Takanori Yamagata, Masami K. Yamaguchi, Ryoichi Sakuta, Ippeita Dan

**Affiliations:** a Institute for Developmental Research, Aichi Developmental Disability Center, Department of Functioning and Disability, Kagiya-cho, Kasugai, Aichi, Japan; b RISTEX (Research Institute of Science and Technology for Society) Group, Kasuga, Bunkyo, Tokyo, Japan; c Jichi Medical University, Department of Pediatrics, Yakushiji, Shimotsuke, Tochigi, Japan; d Chuo University, Applied Cognitive Neuroscience Laboratory, Kasuga, Bunkyo, Tokyo, Japan; e International University of Health and Welfare, Department of Pediatrics, Iguchi, Nasushiobara, Tochigi, Japan; f Dokkyo Medical University, Child Development and Psychosomatic Medicine Center, Minamikoshigaya, Koshigaya, Saitama, Japan; g Chuo University, Research and Development Initiative, Kasuga, Bunkyo, Tokyo, Japan; h Jichi Medical University, Yakushiji, Shimotsuke, Tochigi, Japan; i Japan Women’s University, Department of Psychology, Nishi-Ikuta, Tama, Kawasaki, Kanagawa, Japan; j Chuo University, Department of Psychology, Higashinakano, Hachioji, Tokyo, Japan

**Keywords:** face processing, facial expressions, attention-deficit hyperactivity disorder, near-infrared spectroscopy, occipito-temporal area

## Abstract

**Significance:** It has been reported that children with attention-deficit hyperactivity disorder (ADHD) have impairment in the recognition of angry but not of happy facial expressions, and they show atypical cortical activation patterns in response to facial expressions. However, little is known about neural mechanisms underlying the impaired recognition of facial expressions in school-aged children with ADHD and the effects of acute medication on their processing of facial expressions.

**Aim:** We aimed to investigate the possibility that acute administration of methylphenidate (MPH) affects processing of facial expressions in ADHD children.

**Approach:** We measured the hemodynamic changes in the bilateral temporo-occipital areas of ADHD children observing the happy and angry facial expressions before and 1.5 h after MPH or placebo administration in a randomized, double-blind, placebo-controlled, crossover design study.

**Results:** We found that, regardless of medication, happy expressions induced increased oxyhemoglobin (oxy-Hb) responses in the right inferior occipital region but not in the superior temporal region. For angry expressions, oxy-Hb responses increased after MPH administration, but not after placebo administration, in the left inferior occipital area, whereas there was no significant activation before MPH administration.

**Conclusions:** Our results suggest that (1) ADHD children consistently recruit the right inferior occipital regions to process happy expressions and (2) MPH administration to ADHD children enhances cortical activation in the left inferior occipital regions when they process angry expressions.

## 1 Introduction

Attention-deficit hyperactivity disorder (ADHD) is one of the most common psychiatric developmental disorders, and it affects ∼10% of school-aged children in the United States.^1^ ADHD is characterized by three core symptoms: inattention, hyperactivity, and impulsivity. Therefore, most research with ADHD children has focused on cognitive functions and its impairments related to these core symptoms (e.g., executive functions, attention, etc.). Recent findings, however, reveal that, in addition to these cognitive impairments, ADHD includes some deficits in social cognition.

One of the most important aspects of social cognition is emotion perception, especially through the processing of facial expressions. Faces convey a wealth of information about a person’s emotional state,^2^ and we use them to successfully communicate socially with other individuals. Yet, it has been reported that 37% of school-aged ADHD children have impairment of emotion recognition.^3^ Atypical processing of facial expressions, especially negative expressions, such as anger, fear, and sadness, has been reported for children with ADHD^4^^–^^14^ and for school-aged children at risk of ADHD,^15^ as well as for adult ADHD patients.^16^^,^^17^ Some previous studies revealed that ADHD children had poor performance in the recognition of angry facial expressions compared with typically developing (TD) children, whereas that of happy facial expressions was not significantly different between ADHD and TD children.^5^^,^^8^^,^^13^ In accordance with this behavioral evidence, neuroimaging studies with ADHD children have found atypical patterns of brain activation during recognition of facial expressions in the temporal areas, which are reported to be crucial for face processing.^5^^,^^18^ Williams et al.^5^ measured event-related potentials (ERPs) while ADHD children observed facial expressions and found that neural responses in ADHD children were significantly different from those in TD children for angry expressions but not for happy expressions. Furthermore, a near-infrared spectroscopic (NIRS) study compared hemodynamic responses with angry and happy expressions between ADHD children and TD children.^18^ ADHD children showed increased hemodynamic responses in the right temporal area for happy expressions but not for angry expressions, while TD children showed increased hemodynamic responses in the right temporal area for both facial expressions.^18^ Previous behavioral and neuroimaging findings suggest that the impaired ability to process facial expressions in ADHD children could be related to an atypical neural processing of facial expressions.

It has been reported that the behavioral and cognitive characteristics of ADHD are partly related to dopamine (DA) and noradrenaline (NA) dysfunctions^19^ and that impaired cognition and social functions improve with the administration of psychostimulant drugs, such as methylphenidate (MPH).^20^ For ADHD children, one of the most common first-choice treatments is the administration of MPH. It is believed that MPH increases synaptic transmission by inhibiting reuptake of catecholamines, mainly DA, and acts as a DA agonist in the cerebral cortices.^21^ Although it is known that MPH affects both the DA and NA systems,^22^^,^^23^ effects on the DA system are far greater than those on the NA system because MPH has an affinity to DA receptors 10 times higher than that to NA receptors.^24^ Recent studies have shown that MPH improves both cognitive performance and cerebral processing during cognitive tasks in ADHD children.^25^^,^^26^ For example, Monden et al.^25^ found that, although ADHD children had less accurate performance and lower activation in the right inferior and middle frontal gyri during a go/no-go task than did TD children before MPH administration, there were no differences in either accuracy or brain activity between ADHD and TD children after MPH administration.

Given that MPH improves neurocognitive processing during go/no-go tasks (e.g., Ref. 25), recognition of facial expressions might also be improved by MPH administration. One previous study investigated the effect of MPH on recognition of facial expressions and found that recognition performance for angry expressions 4 weeks after treatment with MPH was higher than that before treatment.^5^ Nonetheless, even after treatment with MPH, ADHD children’s ability to recognize angry expressions was still significantly lower than that of TD children. This finding suggests that ADHD children’s impaired recognition of facial expressions results from dysfunctions in the core cortical regions involved in the processing of facial expressions, such as the superior temporal sulcus (STS), as well as the fusiform gyrus (FG) and the inferior occipital gyrus (IOG),^27^ and that in ADHD children, an alternative or compensating processing of angry expressions may be driven as a result of MPH administration.

To investigate the possibility that acute administration of MPH affects processing of facial expressions in ADHD children, we used functional near-infrared spectroscopy (fNIRS) to measure cortical hemodynamic responses while ADHD children observed facial expressions in a randomized, double-blind, placebo-controlled, crossover design study. We compared hemodynamic responses in ADHD children’s temporal and occipital areas during the presentation of angry and happy facial expressions both before and after MPH or a placebo administration. Considering previous evidence that MPH administration improves recognition of angry expressions,^5^ we predicted that cortical areas involved in the recognition of angry expressions might exhibit increased activation after MPH administration.

## 2 Materials and Methods

### 2.1 Participants

The final sample of participants for this study consisted of 19 clinically referred right-handed Japanese children (one female), who were diagnosed as ADHD based on the DSM-5^28^ by trained pediatric neurologists and required administration of MPH (mean age=9.84 years, SD=1.26  years, range 8 to 12 years; Table 1). An additional six ADHD boys participated but were excluded from the statistical analysis because of an insufficient number of viable trials due to failure to look at the face stimuli for more than three trials for either the happy or angry expression condition or due to motion artifacts. The full-scale IQ scores of participants were assessed using the Wechsler Intelligence Scale of Children Third (WISC-III) or Fourth (WISC-IV) and were all above 70 (mean=92.68, SD=14.0, range 74 to 129). All participants had been taking MPH (18 to 45  mg/day) as part of their regular medication regimen. Specific acute doses were the same as the patients’ daily doses. The diagnosis of comorbid psychiatric conditions (e.g., autism spectrum disorder) was also established by experienced pediatric neurologists based on the DSM-5.^28^

**Table 1:** Demographic and clinical profiles

ID	Age (years)	Sex	ADHD subtype	Complication	MPH dose (mg)	WISC III or IV Full-IQ	Duration of MPH exposure (months)	Other medication
1	10	Female	Combined	None	45	74	2.8	Levocetirizine hydrochloride, magnesium oxide
2	12	Male	Inattentive	None	27	80	1.0	None
3	9	Male	Combined	None	18	84	0.5	None
4	12	Male	Inattentive	None	27	85	2.2	None
5	10	Male	Combined	None	18	94	2.3	None
6	8	Male	Combined	None	18	92	0.2	None
7	11	Male	Combined	ASD	18	120	2.3	Aripiprazole
8	8	Male	Combined	ASD	27	105	0.6	Aripiprazole
9	11	Male	Combined	None	27	76	2.1	None
10	10	Male	Inattentive	ASD	18	129	0.7	None
11	10	Male	Combined	None	27	85	1.9	Aripiprazole
12	11	Male	Combined	None	27	86	1.9	None
13	8	Male	Combined	ASD	27	96	2.2	None
14	10	Male	Inattentive	Type II diabetes, morbid obesity, hyperlipidemia	45	97	2.6	None
15	11	Male	Combined	None	27	82	2.6	None
16	9	Male	Combined	ASD	18	99	1.5	None
17	9	Male	Combined	None	18	85	3.4	None
18	9	Male	Combined	None	27	99	1.6	None
19	9	Male	Combined	ASD	18	93	2.0	None
Mean	9.8					92.7		
SD	1.3					14.0		

All participants and their parents gave oral consent to participate in the study and written informed consent was obtained from the parents of all participants. The study was approved by the Ethics Committees of Jichi Medical University Hospital. The experiments were conducted in accordance with the latest version of the Declaration of Helsinki.

### 2.2 Stimuli

The same stimuli were used as in previous fNIRS studies.^18^^,^^29^ The stimuli consisted of full-color photographs of neutral, happy, and angry facial expressions of five Japanese females. The size of the face stimuli was ∼13×10  deg in visual angle. For the baseline, we presented a blank screen.

There were two test conditions: the happy expression condition and the angry expression condition. For both the happy and angry conditions, one of the five females was chosen randomly for each trial. The sequence of stimulus presentation was identical to that in the previous study.^18^ We presented an image of a neutral face for 400 ms followed by a happy or angry expressions for 400 ms, which allowed participants to perceive the presented faces as having dynamic expressions that changed from neutral to happy or from neutral to angry. The 200-ms interstimulus interval was filled with a fixation point (black dot; 3.5×3.5  deg in visual angle). Each trial lasted 10 s and followed a baseline period of at least 20 s. The duration of the baseline period was controlled by an experimenter. To draw and retain the children’s attention, fixation points during both the baseline and the test were accompanied by respective beeping sounds presented at 1 Hz.

### 2.3 Procedure

Each participant was tested while sitting in a chair and looking at a computer screen ∼40  cm away. Participants observed the face stimuli passively while their brain activity was measured. During measurements, we monitored the participants’ behavior to evaluate the validity of each trial.

### 2.4 Experimental Design

**Fig. 1:**
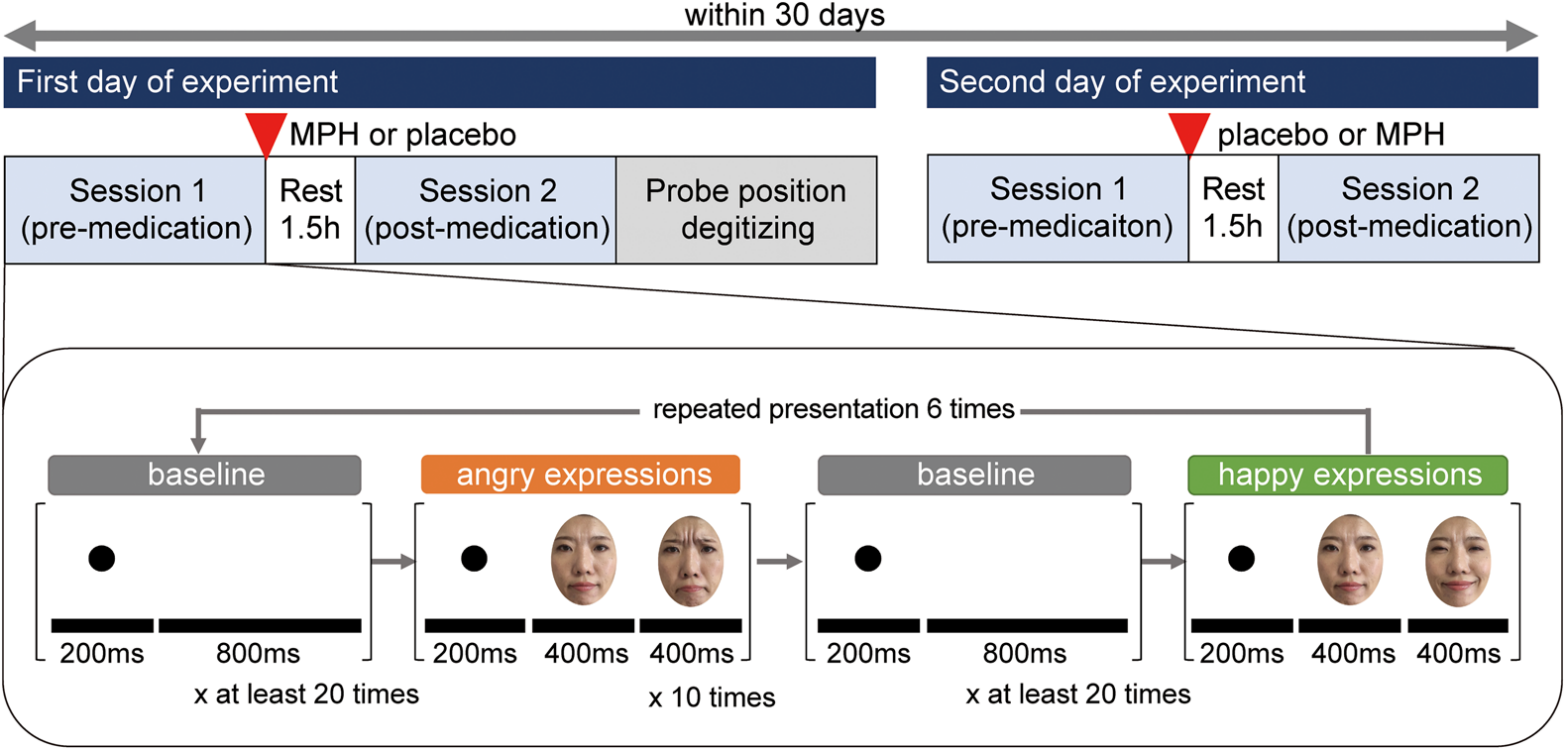
Experimental design. A schematic showing the flow of a pre- and postmedication administration session.

The effects of MPH were examined in a randomized, double-blind, placebo-controlled, crossover study while participants observed happy and angry facial expressions (Fig. 1). We investigated ADHD participants twice within 30 days: one day for medication with MPH and the other day for medication with a placebo. The order of medication of MPH or a placebo was pseudorandomized across participants to avoid order effects.

On each day, ADHD participants underwent two sessions, one before administration of MPH or placebo and the other at 1.5 h after administration. Before each premedication session, all participants underwent an MPH washout period of 4 days. According to the previous study,^18^ each session consisted of 12 trials (6 trials for the happy expression condition and 6 for the angry expression condition). The order in which the two facial expressions were presented (happy expressions first or angry expressions first) was counterbalanced across participants and was the same across all four sessions for each participant.

After measurement of the first session, either MPH (OROS-MPH or Concerta) or a placebo was administered orally. Specific acute doses were the same as the participants’ daily doses, as shown in Table 1.

### 2.5 fNIRS Measurements

We used the multichannel fNIRS system ETG-4000 (Hitachi corporation, Tokyo, Japan) with two wavelengths of near-infrared light (695 and 830 nm) and two sets of fNIRS probes (3×5 array) to measure oxygenated hemoglobin (oxy-Hb) and deoxygenated hemoglobin (deoxy-Hb) signal changes from 44 channels with a sampling rate of 10 Hz. We analyzed optical data based on the modified Beer–Lambert’s law,^30^ as previously described.^31^ Oxy-Hb and deoxy-Hb signal changes were calculated in units of millimolar-millimeter (mM-mm).^31^^,^^32^

Each fNIRS probe contained 15 optical fibers with eight emitters and seven detectors, which were arranged alternately with an emitter–detector distance of 3 cm. Each pair of adjacent emitting and detecting fibers was defined as a single measurement channel, resulting in 22 channels (Ch) for each probe.

We set the fNIRS probes at the bilateral temporal areas because these regions have been reported as important regions for face processing (e.g., Refs. 33 and 34). The probes in the present study covered the probe placements by Ichikawa et al.^18^ After positioning of the probes, the experimenter confirmed whether the fibers were touching each participant’s scalp correctly. If inadequate contact between the fibers and the participant’s scalp was detected, channels were excluded from statistical analysis.

After measurement, we obtained positional data for emitters, detectors, and reference points (nasion: Nz, midline central: Cz, and left and right preauricular points) in real-world space using a 3D digitizer (Eastscan, Polhemus). To identify the spatial positions of measurement channels, we adopted the probabilistic registration method^35^^–^^37^ for registering fNIRS data to Montreal Neurological Institute (MNI) standard brain space, as described in previous studies (e.g., Ref. 26). Spatial profiles of the channels where activation was observed during presentation of facial expressions are shown in Table 2.

**Table 2:** Spatial profiles of the channels activated for facial expressions

		MNI coordinates x, y, z (SD)	Macroanatomy	Prob.	BA	Prob.
Left	Channel 18	−52, −78, −10 (14)	Left inferior occipital gyrus	0.63	19-V3	0.70
			Left middle occipital gyrus	0.25	18-V2	0.21
					37-FA	0.09
Right	Channel 4	58, −65, −22 (14)	Cerebellum crus	0.51	37-FA	0.52
			Right inferior temporal gyrus	0.40	19-V3	0.38
			Right inferior occipital gyrus	0.05	20-Inferior temporal gyrus	0.11
	Channel 8	67, −55, −4 (12)	Right middle temporal gyrus	0.60	37-FA	0.50
			Right inferior temporal gyrus	0.40	21-Middle temporal gyrus	0.49
	Channel 9	51, −80, −10 (14)	Right inferior occipital gyrus	0.58	19-V3	0.61
			Right inferior temporal gyrus	0.13	18-V2	0.37
			Right middle occipital gyrus	0.11		
	Channel 10	62, 17, 23 (14)	Right inferior frontal operculum	0.37	45-pars triangularis Broca’s area	0.34
			Right inferior frontal triangularis	0.29	9-Dorsolateral prefrontal cortex	0.32
			Right precentral gyrus	0.28	44-Pars opercularis, part of Broca’s area	0.23
	Channel 13	59, −70, 7 (14)	Right middle temporal gyrus	0.72	19-V3	0.41
			Right middle occipital gyrus	0.16	37-FA	0.24
			Right inferior temporal gyrus	0.11	39-Angular gyrus, part of Wernicke’s area	0.23
	Channel 15	66, −2, 36 (14)	Right postcentral gyrus	0.63	43-Subcentral area	0.40
			Right precentral gyrus	0.37	6-Premotor and supplementary motor cortex	0.82
					9-Dorsolateral prefrontal cortex	0.12

Note: Prob., probability that a given channel is located on an indicated cortical structure; SD, standard deviation.

### 2.6 Data Analysis

Before analyzing the data, we evaluated the validity of each trial. We excluded a trial from analysis if (a) participants did not look at the face stimuli or (b) body movements were detected based on the experimenter’s visual examination during measurements. In the current study, no channels were rejected for either happy expression or angry expression conditions.

We analyzed oxy-Hb and deoxy-Hb concentrations for further analysis. Individual time-course data for the oxy-Hb and deoxy-Hb changes of each channel were preprocessed with a first-degree polynomial fitting and bandpass filter using cut-off frequencies of 0.01 to 0.8 Hz to remove baseline drift or noise from heartbeat pulsations. Based on the preprocessed time series data, channel-wise and participant-wise contrasts were obtained for each channel by calculating the intertrial mean of differences between the oxy-Hb or deoxy-Hb changes for tests (from 5 to 15 s after face stimulus onset) and baseline (3 s before face stimulus onset). We removed the trials if sudden, obvious, and sharp changes were detected in the time courses of oxy-Hb changes based on independent visual examination.

### 2.7 Statistical Analysis

We performed statistical analyses for the oxy-Hb and deoxy-Hb signals of each channel. We calculated the following contrasts: (1) averaged premedication contrast: averaged activation between preplacebo and pre-MPH versus baseline, (2) postmedication contrasts: face stimuli versus baseline activations for postplacebo and post-MPH, (3) intramedication contrast: difference between post- and premedication activations for each medication (i.e., “postplacebo–preplacebo” and “post-MPH–pre-MPH”), and (4) intermedication contrast: difference between intra-MPH and intraplacebo contrasts (i.e., “post-MPH–pre-MPH” versus “postplacebo–preplacebo”). For all contrasts, we performed two-tailed one-sample t-tests against zero with an effective multiplicity ($M_{\mathrm{eff}}$) correction method^38^ to correct familywise error. In $M_{\mathrm{eff}}$ correction, fNIRS data were obtained from 44 channels (22 channels for each hemisphere) for 19 participants in each condition, and summary data for analyses were denoted as $\beta^{44\;\times \;19}$. Using the eigenvalues derived from a correlation matrix (44×44) of the measured signals ($\beta^{44\;\times \;19}$), the $M_{\mathrm{eff}}$ value was calculated for each contrast for each facial expression (angry and happy). The statistical significance level of 0.05 was divided by each $M_{\mathrm{eff}}$ value.

## 3 Results

We obtained hemodynamic responses from 19 ADHD children who observed face stimuli for more than three trials for both happy and angry expression conditions. The mean number of trials was 5.05 (SD=0.91) for happy expressions and 4.95 (SD=1.13) for angry expressions before MPH administration, and 4.89 (SD=1.05) for happy expressions and 4.89 (SD=1.05) for angry expressions after MPH administration. Before placebo administration, the mean number of trials was 4.89 (SD=0.94) for happy expressions and 5.05 (SD=0.91) for angry expressions, and it was 5.05 (SD=0.85) for happy expressions and 5.21 (SD=0.92) for angry expressions after placebo administration. We conducted a three-way repeated-measures analysis of variance (ANOVA) on the number of trials with medication (MPH versus placebo), session (pre versus post), and condition (happy versus angry) as a within-subject factor and found no significant main effect or interaction. The mean number of valid trials in the current study was almost the same as that in the previous study.^18^

### 3.1 Analyses of Oxy-Hb Signals

**Fig. 2:**
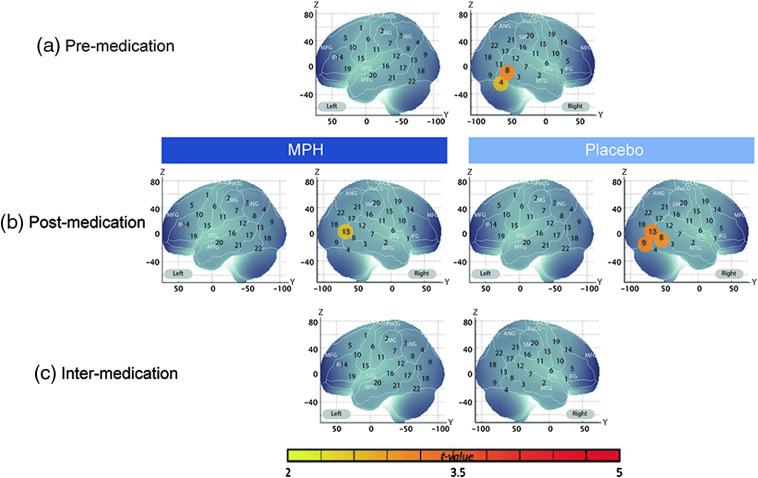
Cortical hemodynamic responses for happy facial expressions before and after MPH and placebo administration. The t-maps of oxy-Hb concentration are shown with significant t-values (two-tailed one-sample t-test with $M_{\mathrm{eff}}$ correction). All coordinates are in MNI space. (a) Premedication contrasts: averaged activation between preplacebo and pre-MPH versus baseline, (b) postmedication contrasts: face stimuli versus baseline for postplacebo and post-MPH, and (c) intermedication contrasts: difference between intra-MPH and intraplacebo contrasts (“post-MPH–pre-MPH” versus “post-placebo–preplacebo”).

**Fig. 3:**
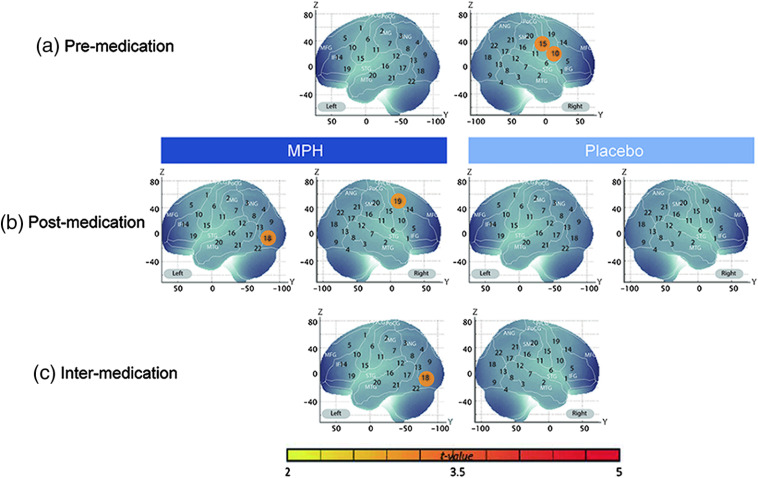
Cortical hemodynamic responses for angry facial expressions before and after MPH and placebo administration. The t-maps of oxy-Hb concentration are shown with significant t-values (two-tailed one-sample t-test with $M_{\mathrm{eff}}$ correction). All coordinates are in MNI space. (a) Premedication contrasts: averaged activation between preplacebo and pre-MPH versus baseline, (b) postmedication contrasts: face stimuli versus baseline for postplacebo and post-MPH, and (c) intermedication contrasts: difference between intra-MPH and intraplacebo contrasts (“post-MPH–pre-MPH” versus “postplacebo–preplacebo”).

Comparison of averaged oxy-Hb concentrations between preplacebo/pre-MPH and a baseline of zero (averaged premedication contrast) revealed that happy expressions induced significant increases of oxy-Hb concentration in the right channel 4 (M=0.031, p<0.01, Cohen’s d=0.69) and the right channel 8 (M=0.025, p<0.01, Cohen’s d=0.78), with an $M_{\text{eff}}$ value of 12.04 [Fig. 2(a) and Table 3]. For angry facial expressions, we found significant increases in the right channel 10 (M=0.045, p<0.01, Cohen’s d=0.81) and the right channel 15 (M=0.041, p<0.01, Cohen’s d=0.81), with an $M_{\text{eff}}$ value of 12.82 [Fig. 3(a) and Table 4].

**Table 3:** Channels showing significant changes in oxy-Hb signals compared with baseline in contrast to happy facial expressions.

		Mean	SD	t	p
Premedication	Right channel 4	0.031	0.05	3.01	0.008*
	Right channel 8	0.025	0.03	3.42	0.003*
Post-MPH	Right channel 13	0.056	0.08	2.98	0.008*
Postplacebo	Right channel 8	0.039	0.05	3.30	0.004*
	Right channel 9	0.068	0.09	3.63	0.003*
	Right channel 13	0.058	0.08	3.38	0.003*
Intra-MPH	—				
Intraplacebo	—				
Intermedication	—				

Note: SD, standard deviation; t, t-value; p, p value.

* Statistically significant (p values are corrected by $M_{\mathrm{eff}}$ correction).

**Table 4:** Channels showing significant changes in oxy-Hb signals compared with baseline in contrast to angry facial expressions.

		Mean	SD	t	p
Premedication	Right channel 10	0.045	0.06	3.53	0.002*
	Right channel 15	0.041	0.05	3.52	0.002*
Post-MPH	Left channel 18	0.051	0.07	3.30	0.004*
	Right channel 19	0.046	0.06	3.24	0.005*
Postplacebo	—				
Intra-MPH	—				
Intraplacebo	—				
Intermedication	Left channel 18	0.058	0.08	3.28	0.004*

Note: SD, standard deviation; t, t-value; p, p value.

* Statistically significant (p values are corrected by $M_{\mathrm{eff}}$ correction).

After medication with MPH, oxy-Hb signal in the right channel 13 increased significantly compared with the baseline (M=0.056, p<0.05, Cohen’s d=0.68) for happy expressions, with an $M_{\mathrm{eff}}$ value of 10.69 [Fig. 2(b) and Table 3], and oxy-Hb signal increased significantly in the left channel 18 (M=0.051, p<0.05, Cohen’s d=0.76) and the right channel 19 (M=0.046, p<0.05, Cohen’s d=0.74) for angry expressions, with an $M_{\mathrm{eff}}$ value of 12.15. [Fig. 3(b) and Table 4]. On the other hand, placebo administration induced significant oxy-Hb activations for happy expressions in the right channel 8 (M=0.039, p<0.05, Cohen’s d=0.76), the right channel 9 (M=0.068, p<0.05, Cohen’s d=0.77), and the right channel 13 (M=0.058, p<0.05, Cohen’s d=0.78), with an $M_{\text{eff}}$ value of 12.8. However, no significant changes in oxy-Hb signal were observed for angry expressions, with an $M_{\text{eff}}$ value of 12.57.

To examine the effects of administering a placebo or MPH, we assessed the intramedication contrast, which is the difference between post- and premedication. We found that oxy-Hb signal after MPH administration was marginally higher than that before MPH administration only for angry faces in the left channel 18 (M=0.044, p<0.10, Cohen’s d=0.66), with an $M_{\mathrm{eff}}$ value of 12.81. Conversely, there was no significant difference for either happy expressions, with an $M_{\mathrm{eff}}$ value of 13.01, or angry expressions, with an $M_{\mathrm{eff}}$ value of 11.87, with placebo administration.

Finally, we analyzed the intermedication contrast to investigate any effect of medication with MPH on oxy-Hb changes during the presentation of angry expressions and happy expressions that was not present with the placebo. We found that the left channel 18 reached significance (M=0.058, p<0.05, Cohen’s d=0.75) for angry expressions, with an $M_{\mathrm{eff}}$ value of 12.20 [Fig. 3(c) and Table 4], but not for happy expressions, with an $M_{\mathrm{eff}}$ value of 12.96 [Fig. 2(c) and Table 3]. These results indicate that MPH, but not the placebo, activated the region around the left IOG when ADHD children observed angry faces.

### 3.2 Analyses of Deoxy-Hb Signals

Tables 5 and 6 represent the channels showing significant changes in deoxy-Hb signals. As for the averaged premedication contrast, which is a comparison of averaged signals between preplacebo/pre-MPH and the baseline of zero, we found no significant changes in any channels for both happy expressions, with an $M_{\mathrm{eff}}$ value of 12.07, and angry expressions, with an $M_{\mathrm{eff}}$ value of 12.52.

**Table 5:** Channels showing significant changes in deoxy-Hb signals compared with baseline in contrast to happy facial expressions.

		Mean	SD	t	p
Premedication	—				
Post-MPH	—				
Postplacebo	Left channel 18	−0.022	0.02	−3.78	0.001*
Intra-MPH	—				
Intraplacebo	Left channel 20	−0.044	0.05	−3.45	0.003*
	Right channel 3	−0.020	0.02	−3.89	0.001*
	Right channel 17	−0.032	0.03	−4.07	0.001*
Intermedication	—				

Note: SD, standard deviation; t, t-value; p, p value.

* Statistically significant (p values are corrected by $M_{\mathrm{eff}}$ correction).

**Table 6:** Channels showing significant changes in deoxy-Hb compared with baseline in contrast to angry facial expressions.

		Mean	SD	t	p
Premedication	—				
Post-MPH	Right channel 15	−0.020	0.02	−3.49	0.003*
	Right channel 19	−0.032	0.04	−3.30	0.004*
Postplacebo	—				
Intra-MPH	—				
Intraplacebo	—				
Intermedication	—				

Note: SD, standard deviation; t, t-value; p, p value.

* Statistically significant (p values are corrected by $M_{\mathrm{eff}}$ correction).

MPH administration induced significant decreases in deoxy-Hb signals for angry expressions in the right channel 15 (M=−0.020, p<0.05, Cohen’s d=0.82) and the right channel 19 (M=−0.032, p<0.05, Cohen’s d=0.78), with an $M_{\mathrm{eff}}$ value of 12.44. We found no significant changes for happy expressions in all channels, with an $M_{\mathrm{eff}}$ value of 9.78. After placebo administration, deoxy-Hb concentrations were significantly decreased in the left channel 18 (M=−0.022, p<0.05, Cohen’s d=0.89) only for happy expressions, with an $M_{\mathrm{eff}}$ value of 12.97, but not for angry expressions, with an $M_{\mathrm{eff}}$ value of 13.59.

For the intramedication contrast, there was no significant difference for both happy expressions, with an $M_{\mathrm{eff}}$ value of 10.74, and angry expressions, with an $M_{\mathrm{eff}}$ value of 12.12, with MPH administration. After placebo administration, we found significant decreases in deoxy-Hb signals in the left channel 20 (M=−0.044, p<0.05, Cohen’s d=0.81), the right channel 3 (M=−0.020, p<0.05, Cohen’s d=0.92), and the right channel 17 (M=−0.032, p<0.05, Cohen’s d=0.96) for happy expressions, with an $M_{\text{eff}}$ value of 13.57. In contrast, no significant changes were found for angry expressions, with an $M_{\text{eff}}$ value of 13.03.

For the intermedication contrast, no channels reached significance either for happy expressions, with an $M_{\text{eff}}$ value of 11.84, or for angry expressions, with an $M_{\text{eff}}$ value of 12.16.

## 4 Discussion

The current study explored whether administration of MPH affects cortical processing of facial expressions in school-aged ADHD children using fNIRS. To this end, we measured hemodynamic responses in temporal and occipital regions during the presentation of angry and happy facial expressions before and after medication with MPH or a placebo. We found that happy facial expressions induced significant increases in oxygenated hemodynamic (oxy-Hb) responses compared with baseline in the right inferior occipital area before and after administration of either a placebo or MPH. On the other hand, angry facial expressions induced no significant increases before MPH administration, whereas increased oxy-Hb responses occurred in the left inferior occipital area after MPH administration. This pattern of brain activation was not found for placebo administration. Importantly, we found that the MPH-induced activation was significantly higher than the placebo-induced activation in the left inferior occipital area in response to angry facial expressions but not to happy facial expressions.

We used the same stimuli and compatible procedures as a previous study that investigated the perception of angry and happy facial expressions in ADHD children,^18^ and our results are consistent with the previous findings. As for the activation with happy expressions, we found that the channels in the right inferior occipital region showed increasing oxy-Hb responses consistently across sessions. Although the left inferior occipital region showed weak oxy-Hb responses to happy expressions before $M_{\text{eff}}$ correction, they did not reach statistical significance. These patterns of brain activation are concordant with the previous findings,^18^ showing a significant increase for happy expressions in the right temporal area but not in the left temporal area (note that channels covering temporal and occipital areas in each hemisphere were investigated in the previous study). For angry expressions, we revealed no significant increases of oxy-Hb signals in any channel except after MPH administration, which is also consistent with the previous study.^18^ Taken together, our results replicated the previous findings reported by Ichikawa et al.^18^

The results showing a significant activation for happy expressions regardless of medication are consistent with previous behavioral findings that ADHD children show comparable recognition performance for happy expressions relative to TD children.^5^^,^^8^^,^^13^ Also, our finding of increased hemodynamic responses in the right hemisphere, but not in the left, is in line with evidence of right hemispheric advantage for processing of facial expressions in adults^39^^–^^42^ and, thus, supports the notion that ADHD children have a preserved ability to process happy facial expressions.

The results we obtained from the activation levels of each channel may provide new insights into the neural processing of facial expressions in ADHD children. In the current study, ADHD children showed significant increases of oxy-Hb signal for happy facial expressions at the channels located in the inferior occipital region of the right hemisphere (Brodmann area (BA) 19: right IOG rather than the superior temporal region. Considering a former study showing that the face area at the IOG [occipital face area (OFA)] is located in BA 18 or 19,^43^ the increased oxy-Hb signal for happy facial expressions may reflect face-specific processing in the right inferior occipital area or on a pathway from the IOG to the STS. It has been reported that face-specific activations are typically observed in the IOG (OFA), the fusiform gyrus (FG) [fusiform face area (FFA)], and the posterior superior temporal sulcus (pSTS face area).^27^ The neural model of face processing proposes that there are two distinct pathways for processing faces: the dorsal pathway (from the OFA to the pSTS) is involved in processing changeable facial information (e.g., facial expressions, eye gaze, and head rotation), whereas the ventral pathway (from the OFA to the FFA) is involved in processing invariant facial information (e.g., identity, race, and age).^34^^,^^44^ Consistent with this neural model, one fMRI study with adults revealed that there was a subnetwork for face processing that includes the left and right STS, which are involved in processing facial expressions, and that the bilateral OFAs play an important role in the face processing. Participants exhibited a significant decrease in functional connectivity between the bilateral OFAs and the right STS when they switched from a face recognition task to an object recognition task.^45^ Additionally, the OFA acts as the first node in this expression subnetwork and projects information about physical form or face parts to the STS,^43^^,^^46^^–^^49^ whereas the pSTS is sensitive to faces which convey valence information.^50^ Given these previous findings and considering the activation pattern exhibiting the inferior occipital region but not superior temporal regions, although ADHD children’s recognition of happy expressions is thought to be intact,^5^^,^^8^^,^^13^ our results suggest that they may actually be relying more heavily on the cortical processing of physical form or facial parts than on the valence conveyed by facial expressions when observing the happy expressions.

A previous finding showing a strong functional connectivity between the bilateral OFAs and the right STS^45^ may also give an account of the significant MPH-induced activation in the left inferior occipital region (left BA 19) while observing angry facial expressions. In contrast to our prediction that MPH administration would induce activation in the right temporal area when observing angry expressions, which was based on previous findings showing a significant activation in the right temporal area in TD children,^18^ we found that MPH, but not a placebo, induced increases of oxy-Hb concentrations in the left inferior occipital area. Our results, however, are consistent with a previous ERP study that reported a significant improvement in the amplitude of the P300 component with MPH around the left posterior temporal region with angry expressions.^5^ One possibility may be that MPH could lead to the promotion of processing in the left occipital inferior region, which is part of the dorsal pathway related to facial expression processing. As mentioned above, both the left and right OFAs have a strong functional connectivity with the right STS.^45^ This suggests that the bilateral OFAs convey physical form information of faces to the right STS for processing of facial expressions. The MPH-induced activation in the left inferior occipital region observed in the current study may reflect that acute MPH administration promotes the processing of the physical form of angry expressions.

The significant increase in oxy-Hb signals with angry expressions induced by MPH administration is probably related to acute modulation of the DA system. Previous studies have shown that the DA system is important for recognition of angry expressions.^51^^,^^52^ For example, a selective disruption in the recognition of angry facial expressions, but not in other facial expressions, occurred in healthy adult participants with acute administration of a DA D2-class receptor antagonist.^51^ This finding implies that, although it is considered that the behavioral and cognitive characteristics of ADHD patients result partly from both DA and NA dysfunctions,^19^ an impairment in the recognition of angry faces in ADHD may be due to the DA dysfunction alone. Given that MPH acts as a DA agonist by blocking DA transporters,^21^ this MPH action could have induced increased oxy-Hb signals when ADHD children observed the angry faces.

In the present study, we found increased oxy-Hb responses in the region around the dorsolateral prefrontal cortex (DLPFC) and premotor cortex only for angry expressions before medication. This pattern of activation is consistent with previous findings that ADHD children exhibit greater activation in the DLPFC for angry expressions compared with TD children^53^ and may be related to emotional impairments in ADHD children. It has been reported that the DLPFC plays an important role in the modulation of aggressive behavior^54^ and regulation of emotional response.^55^^,^^56^ The premotor cortex is activated for perception of whole-body expressions of emotion^57^ and is involved in action preparation and execution.^58^^,^^59^ In addition, a previous study implied that activation in the premotor cortex when observing angry expressions may reflect autonomic reactions and motor responses related to defensive behaviors.^60^ Considering these previous findings, the increases in hemodynamic responses for angry expressions in the prefrontal and premotor areas may be related to evidence that ADHD children have more emotional impulsiveness, defined by quickness to anger, easy emotional excitability, low frustration tolerance, etc., than do control children.^61^

We found that, during passive observation of happy expressions, a slightly broader area of the right inferior occipital region activated after placebo administration (right channels 8, 9, and 13) compared with before placebo administration (right channels 4 and 8) in oxy-Hb signals. This pattern was also found in deoxy-Hb signals: no channel activated before placebo administration, while the left channel 18 activated after placebo administration. Previous EEG studies with psychostimulants^62^ and antidepressants^63^ reported a placebo effect in ∼30% of patients. Also, a placebo effect is occasionally observed within series of studies using the same double-blind, placebo-controlled, crossover-design experiments.^25^^,^^64^^,^^65^ In this respect, our results showing a broader activation after placebo administration may be partly due to a placebo effect, although the neural mechanisms behind this observation are still under debate.^66^

As opposed to oxy-Hb signals, we did not find significant changes in deoxy-Hb signals even for happy expressions in occipital temporal areas across sessions after multiplicity correction (some channels showed significant decreases before multiple correction). These results suggest that changes in deoxy-Hb signals would be a less sensitive indicator of cortical processing of facial expressions than changes in oxy-Hb signals. Our results are consistent with previous findings that the signal amplitude of oxy-Hb is higher than that of deoxy-Hb^67^ and that oxy-Hb is more sensitive to activation related to the processing of facial expressions.^18^^,^^29^ Also, Hoshi and Tanji^68^ mentioned that oxy-Hb is the most sensitive indicator of changes in regional brain activation. Alternatively, deoxy-Hb signals are known to often exhibit delayed responses compared with oxy-Hb signals,^69^ which might not have been well detected in the current experimental design. Considering these findings, our results imply that oxy-Hb signals are more appropriate as an index to evaluate cortical processing of facial expressions than deoxy-Hb signals.

With the exception of the right channel 19 for the angry expressions in a comparison between pre-MPH and post-MPH administration, most channels showing significant increases in oxy-Hb signals did not show significant decreases in deoxy-Hb signals. Although increases in oxy-Hb signals are considered to be accompanied by decreases in deoxy-Hb signals with regional brain activation, deoxy-Hb signals do not necessarily show this pattern of changes.^68^ For example, an increase or absence of changes in deoxy-Hb signals was also observed with increases in oxy-Hb.^70^^–^^72^ Thus, the pattern of changes in deoxy-Hb signals and the applicability of the deoxy-Hb parameter should be carefully evaluated and discussed in further exploration.

We will also discuss a few limitations of the current study. First, we could not provide evidence for how different regions of bilateral temporal areas contribute to the processing of facial expressions in TD children because we did not measure the brain activity of TD children for comparison. Second, we included ADHD children with and without comorbid autism spectrum disorders (ASD). In this study, participants included 13 ADHD children without comorbid ASD and 6 ADHD children with it. A recent fNIRS study implies that ADHD with ASD is characterized by a different neurofunctional pathology than that without ASD.^26^ Given this finding, our sampling may have passively brought a contamination effect from the comorbidity of ASD on the present results.

To investigate the possible effect of ASD comorbidity, supplemental analyses were performed on the oxy-Hb data of ADHD children without comorbid ASD (n=13). We found almost the same tendency as with the full participant set, namely, significant increases in oxy-Hb signals only for angry expressions after MPH administration in the left inferior occipital area (left channel 18) (M=0.058, p<0.05, Cohen’s d=0.99), with an $M_{\mathrm{eff}}$ value of 9.43. Importantly, the tendency of a medication effect induced not by a placebo but by MPH was also observed in the left inferior occipital area only for the presentation of angry expressions: MPH-induced activation was significantly higher than placebo-induced activation before correction for multiple comparisons (M=0.048, p=0.04), while it did not reach significance after $M_{\mathrm{eff}}$ correction with a value of 9.32. Similarly, in the other contrasts, we found a tendency of oxy-Hb increases in the channels showing significant increases in the full participant set. Thus, we consider that the any contamination effect would be small in the current study. However, these points can be tested with future research to extend the current findings.

## 5 Conclusions

In conclusion, the current fNIRS study investigating the possibility that acute administration of MPH affects the cortical processing of facial expressions in ADHD children revealed different modulations in cortical activation patterns in response to the presentation of angry and happy expressions before and after MPH administration. We revealed that ADHD children show increased activation in the right inferior occipital region for happy expressions regardless of MPH or a placebo administration. In contrast, for angry expressions, significant activation was found in the left inferior occipital area after MPH administration but not before MPH administration. Importantly, we found significant MPH-induced, but not placebo-induced, increases in hemodynamic response for angry expressions in the left inferior occipital region, suggesting that MPH promotes the processing of physical forms or facial parts, but not valence information, of angry facial expressions.
